# Chronic disorders, work-unit leadership quality and long-term sickness absence among 33 025 public hospital employees

**DOI:** 10.5271/sjweh.4036

**Published:** 2022-10-01

**Authors:** Amar J Mehta, Jimmi Mathisen, Tri-Long Nguyen, Reiner Rugulies, Naja Hulvej Rod

**Affiliations:** 1 Department of Public Health, University of Copenhagen, Copenhagen, Denmark; 2 National Research Centre for the Working Environment, Copenhagen, Denmark; 3 Copenhagen Stress Research Center, Copenhagen, Denmark

**Keywords:** absenteeism, chronic disease, longitudinal study, managerial quality, occupational health, psychosocial work resource

## Abstract

**Objective:**

This study aimed to examine the association between work-unit level leadership quality and individual-level long-term sickness absence (LTSA) in the hospital sector and effect modification by chronic disorders.

**Methods:**

This longitudinal analysis included 33 025 Danish public hospital employees who were followed-up for one year after baseline in March 2014. Leadership quality was assessed by questionnaire with mean responses aggregated by work-unit and characterized in tertiles. LTSA during follow-up was determined from employer records. Chronic disorders at baseline was assessed from the Danish hospital and prescription registers. We performed multilevel logistic regression to estimate odds ratios (OR) and 95% confidence intervals (CI) adjusting for potential confounders. We evaluated interaction between chronic illness and low leadership quality on multiplicative and additive scales.

**Results:**

We identified employees as healthy (60.8%) or with somatic (31.6%), mental (3.3%), or both somatic and mental (4.3%) disorders. During follow-up, 6% of employees registered a LTSA. Medium and high leadership quality were associated with lower risk of LTSA with OR of 0.84 (95% CI 0.76–0.94) and 0.73 (95% CI 0.65–0.82) respectively, compared to low leadership quality. Associations were similar for healthy employees and employees with only somatic disorders, whereas no association was observed for employees with mental disorders (in presence or absence of somatic disorders). No statistically significant (α=0.05) interactions between leadership quality and chronic disorders on LTSA were observed.

**Conclusion:**

The findings suggest that the quality of leadership in work units is associated with risk of long-term sick leave in the Danish public hospital sector and that strong leadership protects employees against LTSA.

The general workforce population is aging; the proportion of older workers aged 55–64 years is increasing and is projected to equal one quarter of the global workforce by 2030 (1). To maintain labor market participation among workers, many high-income countries are regulating pension schemes to increase the retirement age which will also lead to a higher proportion of older-aged individuals, and those with chronic health conditions or disorders, in the work force (2). In Denmark, approximately 35% of workers in 2013 were reported to have at least one chronic disorder (3).

Workers with chronic disorders generally have a weaker connection to the labor market. Studies in Europe including Denmark have shown that workers with chronic disorders have lower employment rates (4) and higher rates of long-term sickness absence (LTSA) (5–7) in comparison with healthy workers. The previous studies examined chronic disorders that included a wide range of chronic somatic and/or mental health conditions (5, 6), some of which may show no visible symptoms (eg, hypertension, certain autoimmune disorders). The importance of workplace resources may prove particularly relevant for workers with chronic disorders. For example, the handling of health-related matters during work and adaptation of work tasks may affect the risk and length of sick leave and retention in the labor market.

Leadership quality (LQ), or quality of management or supervision, may be considered a workplace psychosocial resource that can help improve labor market participation among workers. LQ refers to specific behaviors or actions of a leader (eg, manager or supervisor) towards their subordinates including the ability to provide guidance, solve conflicts, plan and organize work, prioritize job satisfaction, or support career development (8, 9). Epidemiologic evidence from single and multi-occupational sector studies support the association between LQ with a range of indicators of labor market participation including retention (10), sickness absence (SA) (11–17), and early exit from the labor market (17, 18). Many of the previous studies relied on self-reported LQ at the employee level only, which may lead to reporting bias should workers with chronic disorders have a higher tendency to report a negative work environment.

Better LQ in an employee’s work unit may be hypothesized to be beneficial for employees with chronic disorders as greater openness and flexibility around needs and better organization, help, and support around work tasks have a positive impact. However, whether LQ is similarly or more or less beneficial for workers with chronic disorders and their participation in the labor market is not known. Only a few population-based studies from The Netherlands and UK examined whether chronic disorders modify the association between psychosocial working conditions and resources, sometimes inclusive of leadership/managerial quality, and labor market participation indicators (6, 19–21), but they either objectively assessed only a few specific chronic diseases or relied on self-reported assessment on a wider scope of chronic disorders.

Countering for the methodologic limitations of previous studies that either examined individual-level LQ or included self-reported or limited scope of chronic disorders, the present study aims to investigate if higher LQ at the work-unit level is associated with lower occurrence of LTSA in a large prospective cohort study of hospital employees in Denmark (22). By leveraging the Danish hospital patient registers and prescription registers for objective assessment on a wide range of chronic somatic and mental disorders in the study population, we also evaluated whether the association between work-unit LQ and LTSA is modified by the presence of chronic somatic or mental disorders.

## Methods

### Study population and design

This longitudinal analysis is nested within the Well Being in HospitAL Employees (WHALE) study, an ongoing prospective observational cohort study including all public hospital employees within the Capital Region of Denmark (23). In brief, 37 720 public hospital employees in the Capital Region were invited for a well-being assessment survey in March 2014, of whom 84% participated. Employees’ information on SA and on sex, institution, department, work-unit and professional sector was obtained from employers’ records. Participants were followed up starting on 1 April 2014 for a maximum of 12 months after the date of participation in the 2014 survey or until termination of employment (ie, early/old-age retirement, leaving the workplace, unemployment), which ever came first. A previous analysis of employees who completed the 2014 WHALE survey showed that 10.5% of employees exited from work within 12 months following the survey (10), thus we did not expand the period of follow-up. Because we aggregated LQ at the work-unit level, employees who did not respond to the questionnaire remained in the study sample if all other inclusion criteria were met. A total of 33 025 employees (87.6%) who were nested in 2272 work units were included in this analysis and had complete information on SA records, work-unit LQ, and employee-level and work-unit characteristics; a detailed flowchart of study participation and inclusion can be found in the online supplementary material (www.sjweh.fi/article/4036, figure S1).

### Work-unit leadership quality

LQ was measured using four questions that were adapted from the Interpersonal Relations and Leadership dimension of the validated Copenhagen Psychosocial Questionnaire II (23) using items from the recognition scale, quality of leadership scale, and social support from supervisor scale, and addressed concepts such as recognition, organization of work, support and prioritization of well-being. These questions begin with “To what degree …” and end with “… is your work recognized and appreciated by your manager?”, “… is your manager good at organizing work in your workplace?”, “… do you get help and support from your manager when you need it?”, and “… would you say that your manager prioritizes job satisfaction?” The response scales were 5-point Likert scale ranging from ‘not at all’ to ‘to a very high degree’. The Cronbach’s alpha coefficient of the four items was 0.91. Individual-level LQ was assessed by calculating the mean of the item responses in percentages and converting these into a scale ranging from 0–100, with higher scores representing higher LQ. Individual-level LQ was recorded as missing if the employee responded to only one of the items. Work-unit LQ was further calculated as the aggregated mean of individual-level LQ within each work-unit, and that value was assigned to all employees within the corresponding work unit. Work-unit LQ was treated as missing if individual-level data were available for <50% of eligible employees at the unit level. Work-unit LQ was characterized as both continuous linear and in tertile categories (low, medium, high).

### Chronic somatic and mental disorders

Employees with chronic disorders, understood as long-term or recurrent illness, are defined as those who were hospitalized with or under continuous treatment with prescription medication for one of 13 chronic diseases (cancer, endocrine diseases including diabetes, neurological diseases, eye and ear diseases, cardiovascular diseases, chronic lung diseases, diseases of the digestive system, diseases of the skin, musculoskeletal diseases, chronic pain, kidney diseases, abdominal diseases, and mental illnesses). The chronic diseases were identified in the Danish registers based on previous work (5, 24) and in accordance with the International Classification of Diseases-10th edition (ICD-10) and Anatomic Therapeutic Chemical Classification System (ATC) codes. All chronic diseases were identified by either ICD-10 diagnosis for at least one hospital patient encounter in the Danish National Patient Registry (25) and the Danish Psychiatric Central Research Register (26), or by ATC codes for at least five prescription reimbursements in the Danish National Prescription Registry (27) within five years prior to the administration of the 2014 survey. Supplementary table S1 summarizes the list of the 13 chronic diseases and their corresponding ICD-10 and ATC codes. We constructed a composite chronic disorder variable consisting of four mutually exclusive categories: (i) healthy (ie, no somatic or mental disorders), (ii) only somatic disorders, (iii) only mental disorders, and (iv) both somatic and mental disorders and mental illness.

### Long-term sickness absence

We obtained employees’ SA information from the employers’ payroll system, where SA including its length was registered on a monthly basis. Consistent with how SA episodes were registered in the payroll system, and with current Danish regulations regarding sickness benefits, LTSA was characterized as a binary variable (yes/no) and defined as a SA episode of ≥29 consecutive days that was initiated within one year following baseline. We also measured LTSA within one year prior to baseline.

### Statistical analysis

Similar to methods described in Török et al (28), we used multi-level logistic regression to model LTSA with a random intercept for work-unit as a function of work-unit LQ, and with the Kenward-Roger degrees of freedom (approximation procedure (PROC GLIMMIX procedure, SAS v9.4). The employers’ payroll system did not provide the exact dates of SA and therefore we used logistic rather than Cox regression. We fit models where work-unit LQ was characterized as continuous (per interquartile range unit increase in work-unit LQ) and as categorical tertile variables (medium, high, low tertile as reference). All models were minimally adjusted for age and sex before including adjustment for part-time work (<37 hours/week), seniority (number of years employed in the Capital region), job classification, work-unit size (total number of employees within work-units), and work-unit proportion of female and part-time employees. All associations were presented as odds ratios (OR) with 95% confidence intervals (CI). To infer the preventive potential of work-unit LQ, we calculated the population attributable fraction (PAF) to estimate the percentage of LTSA cases attributable to the exposure to the low and medium tertiles of work-unit LQ (29).

We evaluated interaction between work-unit LQ and chronic disorders on the multiplicative scale by including interaction terms between low and medium tertile categories of work-unit LQ and chronic illness indicators in the model; we set the significance level for interaction terms at P < 0.05. We also assessed additive interaction by calculating the relative excess risk due to interaction (RERI) (30) in the doubly adverse-exposed groups for only somatic illness (i.e. only somatic illness present, low tertile LQ) and mental illness (mental illness present, low tertile LQ). For ease of interpretation, the common reference category in this model was the subgroup of healthy employees in the high tertile of LQ.

As a sensitivity analysis, we examined the influence of prior LTSA as a potential confounding with two different analytical approaches. First, we included prior LTSA as an additional adjustment factor in the logistic regression model. Second, we restricted the sample to employees with no indication of prior LTSA. To address confounding by individual-level socioeconomic status, we also adjusted for household income and marital status, both variables derived from national registries. We additionally assessed the overall interaction between work-unit LQ and individual-level occupational groups to determine if the association between work-unit LQ and LTSA was consistent across occupational groupings. Finally, approximately 6.4% (N=2408) of all invited study participants were excluded from the analysis due to having missing information on either LTSA during following or work-unit LQ. We observed differences in the distributions of individual-level and work-unit characteristics between the excluded subset and the analysis sample (supplementary table S2), and we applied inverse probability weights to account for the potential selection bias arising from this specific exclusion from the analysis sample (31).

## Results

### Baseline characteristics

Table 1 summarizes the employee-level and work-unit-level characteristics of the 33 025 study participants at baseline as a pooled sample and by work-unit LQ tertiles. During the 1-year follow-up period, 6% of employees had a registered LTSA; 7.0%, 5.8% and 5.1% had a registered LTSA during follow-up among employees in the low, medium and high tertiles of work-unit LQ, respectively. Overall, we observed relatively minor differences in the distribution of individual and work-unit-level variables between work-unit LQ tertile subgroups. However, work-unit LQ was inversely correlated with work-unit size. Supplementary figure S2 illustrates the distribution of LQ across work-units. The intraclass coefficient (ICC) for individual-level LQ at the work-unit level was 26% (ICC=0.261), indicating a moderate degree of clustering of LQ *within* work-units.

**Table 1:** Distribution of individual and work-unit level characteristics at baseline in pooled sample and by work-unit leadership tertile. [SD=standard deviation; LTSA=long-term sickness absence].

Characteristics	Total(N=33 025 employees)			Work-unit leadership quality tertile								

				Low (N=10 991 employees)			Medium (N=11 008 employees)			High (N=11 026 employees)		
			
	Median	%	Mean (SD)	Median	%	Mean (SD)	Median	%	Mean (SD)	Median	%	Mean (SD)
Median work-unit leadership quality score	69.0			56.3			68.9			78.7		
Prior long-term sickness (12 months)		4.9			5.8			4.7			4.1	
LTSA during follow-up (12 months)		6.0			7.0			5.8			5.1	
Female		77.8			76.7			77.8			78.8	
Part-time		36.7			36.6			35.8			37.7	
Job classification												
Doctors and dentists		11.8			10.5			13.4			11.6	
Nurses and nursing assistants		40.9			38.8			41.4			42.4	
Other healthcare		15.5			16.9			15.6			14.2	
Education-related staff		2.4			2.5			2.3			2.3	
Service and information technology		11.0			12.7			9.0			11.2	
Administrative staff		18.4			18.6			18.3			18.3	
Age (years)			45.6 (11.3)			45.5 (11.3)			45.3 (11.4)			45.9 (11.2)
Seniority (years)			10.6 (10.0)			10.7 (10.1)			10.2 (9.7)			10.9 (10.0)
Work-unit size			25.6 (19.4)			29.6 (19.4)			26.4 (17.7)			20.8 (13.1)
% Work-unit female			77.7 (25.6)			76.5 (27.9)			77.7 (24.8)			78.7 (24.0)
% Work-unit part-time			35.8 (28.6)			35.7 (28.1)			35.0 (27.8)			36.6 (29.7)

At baseline, we identified 20 081 (60.8%), 10 443 (31.6%), 1090 (3.3%) and 1411 (4.3%) of employees as healthy or with only somatic disorders, mental disorders, or both somatic and mental disorders, respectively (supplementary table S3). Of employees with both somatic and mental disorders, 12.8% had prior LTSA. The respective figures for those with only mental or somatic disorders were 8.2% and 7.6%, considerably higher than that of healthy employees (2.7%). A similar pattern was observed for employees who registered a LTSA during follow-up. Across the major chronic disorder subgroups, highest mean age and years of seniority was observed for employees with only somatic disorders. Employees with mental disorders (with or without somatic disorders) were most often female and part-time employees, and had the highest mean work-unit size and proportions of female and part-time employees in work-units. The subgroups of mental disorders were also very similar in distribution with respect to most individual-level and work-unit characteristics. The distributions of work-unit characteristics were similar between the healthy and only somatic disorder subgroup of employees. Overall, the distribution of job classifications was similar across the major chronic disorder subgroups. Among employees with only chronic somatic disorders, the most occurring subtypes were cardiovascular-related diseases (13.9%), musculoskeletal disorders (12.3%) and chronic pain (8.9%) (supplementary table S4).

### Work-unit LQ and LTSA

Per interquartile range (15.6) increase in work-unit LQ score was associated with a 15% lower odds of LTSA during follow-up (OR 0.85, 95% CI 0.80– 0.90) after adjustment for age, sex, and employee and work-unit level factors (table 2). Similar associations were observed for work-unit LQ in the medium (OR 0.84, 95% CI 0.76–0.94) and high (OR 0.73, 95% CI 0.65–0.82) tertiles in comparison with the low tertile (P_trend_<0.0001). The protective associations between work-unit LQ and LTSA in the linear and categorical models were mildly attenuated after either additional adjustment for prior LTSA or restriction to employees without prior LTSA, but remained statistically significant (table 2).

**Table 2:** Associations^a^ between work-unit leadership quality and the risk of individual-level long-term sickness absence (LTSA) during 1-year follow-up [CI=confidence interval; IQR=interquartile range; OR=odds ratio]

Work-unit leadership quality	Age and sex adjusted		Multiple adjusted ^b^		Multiple adjusted + prior LTSA		Multiple adjusted excluding prior LTSA ^c^	
			
	OR	95% CI	OR	95% CI	OR	95% CI	OR	95% CI
Continuous variable								
Scaled per IQR increase (15.6 units)	0.82	0.78–0.87	0.85	0.80–0.90	0.88	0.83–0.94	0.88	0.82–0.94
Categorical variable								
Tertile 1 (Low)	1.00		1.00		1.00		1.00	
Tertile 2 (Medium)	0.82	0.73–0.91	0.84	0.76–0.94	0.88	0.78–0.98	0.87	0.77–0.99
Tertile 3 (High)	0.71	0.63–0.79	0.73	0.65–0.82	0.77	0.69–0.87	0.76	0.66–0.86

a As estimated in multilevel logistic regression with random intercept for work-unit.

b Multiple adjusted model includes additional adjustment for individual-level part-time status, job seniority, and job classification, and work-unit level unit size, proportion of female employees, and proportion of part-time employees.

c Multiple adjusted model is restricted to employees without LTSA prior to baseline survey.

### Interaction between chronic disorders and work-unit leadership quality

Given the considerably higher prevalence of prior LTSA among employees with somatic and/or mental disorders in comparison with healthy employees, we evaluated the associations between joint categories of major chronic disorders and work-unit LQ tertiles on LTSA during follow-up, and the association between work-unit LQ tertiles and LTSA during follow-up within strata of chronic disorders, among employees *without* prior LTSA (table 3). Lastly, we combined subgroups of only mental disorders and both somatic/mental disorders due to the small number of cases in both groups across strata of work-unit LQ tertiles. Findings from the joint-category models indicate that employees with only somatic disorders and low tertile LQ (ie, doubly adverse exposed) had a 2.37-times higher odds of LTSA (95% CI 1.93–2.90) than the non-exposed employees (ie, healthy and high tertile LQ). The effect size of this joint exposure was higher than expected from the sum of their individual main effects (RERI >0), indicating a supra-additive interaction, but that was not statistically significant as the CI overlapped the null value of 0 (RERI: 0.31, 95% CI -0.14–0.76). Employees with mental disorders and low tertile LQ had a 3.31-times higher odds of LTSA (95% CI 2.50–3.40), but the effect size of the joint exposure category was less than expected from the sum of their individual main effects (RERI <0), indicating a sub-additive interaction, but that was not statistically significant (RERI: -0.48, 95% CI -1.67–0.704).

**Table 3:** Association between joint categories of work-unit leadership quality and chronic disorder and risk of long-term sickness absence (LTSA) with a common reference category, and between work-unit leadership quality and LTSA within strata of chronic disorder, among employees without LTSA in the 12 months prior to the baseline survey. [CI=confidence interval; OR=odds ratio.]

Chronic disorder subgroup	Work-unit leadership quality level	LTSA ^a^ cases / number of subjects	Model with joint categories of leadership quality and chronic disorder ^b^		Models stratified by chronic disorder ^b^	
	
			OR	95% CI	OR	95% CI
Mental disorder	Low	78/783	3.31	2.50–4.40	1.00	0.71–1.40
Mental disorder	Medium	77/729	3.67	2.76–4.87	1.01	0.73–1.42
Mental disorder	High	72/719	3.43	2.57–4.58	1.00	
Somatic disorder only	Low	245/3489	2.37	1.93–2.90	1.38	1.13–1.68
Somatic disorder only	Medium	207/3653	1.90	1.54–2.34	1.11	0.91–1.37
Somatic disorder only	High	192/3777	1.68	1.36–2.08	1.00	
Healthy	Low	235/6082	1.37	1.12–1.68	1.41	1.15–1.73
Healthy	Medium	204/6114	1.21	0.98–1.48	1.23	1.00–1.52
Healthy	High	173/6075	1.00		1.00	

a One or more episodes of ≥29 consecutive days of sickness absence during 1-year follow-up.

b As estimated in multilevel logistic regression with random intercept for work-unit and includes adjustment for individual-level age, sex, part-time status, job seniority, and job classification, and work-unit level unit size, proportion of female employees in work-unit, and proportion of part-time employees in work-unit; the common reference category of joint-exposures was healthy-high tertile work-unit leadership quality.

^c^ As estimated in multilevel logistic regression with random intercept for work-unit and includes adjustment for individual-level age, sex, part-time status, job seniority, and job classification, and work-unit level unit size, proportion of female employees in work-unit, and proportion of part-time employees in work-unit; multilevel logistic regression models performed within separate strata of chronic disorder subgroup.

The association between work-unit LQ tertiles and LTSA within strata of chronic disorder subgroups illustrates that the associations for low tertile exposure were very similar between the healthy and only somatic disorders (table 3); the association for the medium tertile was slightly weaker in magnitude in the only somatic disorder subgroup and did not reach statistical significance. In contrast, associations approximating close to null were identified for employees with any mental disorders. Pairwise multiplicative interactions between chronic disorder types and work-unit LQ tertiles were not statistically significant.

### Population attributable fraction for work-unit leadership quality

Given that we did not observe a statistically significant interaction between chronic disorder and work-unit LQ on either the multiplicative or additive scale, we calculated the PAF for the pooled sample based on the multiple adjusted model where high tertile work-unit LQ is the reference category. The PAF associated with being in low and medium tertiles of unit-level LQ was 15.0%. This means that approximately 297 of the total number of LTSA cases in 1 year (1978) could be prevented if LQ in all work units is raised to the level that is observed in the high tertile, under the assumption that the association between LQ and LTSA is causal.

### Sensitivity analyses

After stratification by job classification, the OR estimates for the medium and high tertiles varied across job classifications, but the CI across job classifications widely overlapped (supplementary table S5); with exception of the specific interaction between high tertile work-unit LQ and education and service/information technology related staff (low tertile work-unit LQ and nurses and nurse assistants as corresponding reference categories), no pairwise interaction terms met statistical significance (P>0.05). The associations between work-unit leadership tertiles and LTSA were robust to additional adjustment for household income and marital status (supplementary table S6), and similarly robust to inverse probability weights for exclusion from the study sample due to missing LTSA status or work-unit-level LQ (supplementary table S7).

## Discussion

The findings from this longitudinal analysis of Danish public hospital employees, which consists of several occupational groups, indicate that higher LQ at the work-unit level was associated with lower risk of individual-level LTSA over a 1-year follow-up period. Assuming this association is causal, approximately 15% of all LTSA cases within one year could be prevented should LQ in work-units be raised to the highest level. While the association between work-unit LQ tertiles and LTSA was similar between healthy employees and employees with only somatic disorders, no association was observed in the small subgroup of employees with mental disorders, who already experienced a high level of LTSA irrespectively of LQ. That said, neither somatic nor mental disorders statistically significantly modified the association between work-unit LQ and LTSA.

While our measure of LQ deviated from the COPSOQ-II’s quality of leadership scale used in many of the previous studies on this topic, the protective association observed for higher work-unit LQ in relation to LTSA is still consistent with several previous studies (11–15, 32, 33). We add new evidence to this finding by demonstrating that the association between LQ and risk of LTSA was similar among both healthy employees and employees with only somatic disorders, whereas the association was absent among employees with mental disorders. There is also limited evidence that is not entirely consistent with the premise that LQ is protective against LTSA. Investigators of the Danish general population 2004–2005 survey-based COPSOQ II study reported a slightly protective but not statistically significant association between LQ and 1-year LTSA (34). Findings from an earlier analysis of the 2000 Danish Work Environment Cohort Study (DWECS) indicate that the stronger protective effect of LQ on 1.5-year LTSA was limited to female workers (35). However a subsequent study, which pooled the COPSOQ II and 2000 DWECS surveys with several other Danish surveys into a large sample of 39 408 workers, found that lower LQ was associated with higher risk of 1.5 year-LTSA (13). Many of the previous studies on LQ and LTSA assessed LQ and LTSA at the individual-level, which may be limited by reporting bias. In a smaller sample (N=1734) of Danish workers in the human services sector, low-level work-unit LQ was associated with 75% higher risk of 1.5-year LTSA (rate ratio: 1.75, 95% CI 1.13–2.38) (15).

We hypothesized that higher work-unit LQ would be more protective against LTSA for employees with pre-existing chronic somatic and mental disorders, so it is notable that the relative association between LQ and LTSA were similar between healthy employees and employees with only somatic disorders, and that no clear association was observed for mental disorders. This indicates that LQ is an important psychosocial work resource both for workers with and without chronic disorders. The lack of association among employees with mental disorders is more puzzling and contrasts with findings from a pooled analysis of the 2000 and 2005 DWECS samples, which showed that higher LQ was more beneficial towards 2-year LTSA for workers with moderate depressive symptoms than for workers with no depressive symptoms (36). A possible explanation is that higher severity of illness and the high rate of LTSA already experienced among employees with mental disorders may make LQ less relevant as an important psychosocial work resource. Also, we focused on severe mental disorders which have led to medication use and/or hospital admission, and it is likely that higher work-unit LQ will be more beneficial or protective against LTSA for workers with early symptoms of mental disorders in comparison with workers with fully developed and clinically diagnosed disorders. The role of selection bias is another possibility through selection of workers with chronic mental disorders out of the study population. In a post-hoc analysis of workers employed during the 2011 wave of WHALE, we observed that workers with chronic mental disorders were less likely to be included in the current study (ie 2014 WHALE), independent of age, sex, and job classification (data not shown). Thus, those who remain employed at the hospital may be more resilient and therefore less responsive to work-unit LQ. Moreover, it is possible that for individuals with mental disorders, who in general have a high risk of LTSA, high work-unit LQ alone might not be sufficient to make a difference with regard to LTSA. Future studies should look into the effects of LQ on retention among people with and without mental disorders to address this issue.

### Strengths and limitations

This study has numerous strengths including a large sample size with a high participation rate, inclusion of a wide range of occupational groups, register-based assessment of chronic disorders, determination of work-units and LTSA from detailed employer records, and the control for of wide array of potential confounders at the employee and work-unit level. By assessing LQ at the work-unit level, we are reducing reporting bias that could be influenced by pre-existing chronic disorders. Similarly, by linking information between chronic disorders (the Danish registers), LQ (questionnaire), and LTSA (employer records) from different data sources, we eliminated common method bias.

There are also limitations to be considered. Baseline measurement of work-unit LQ may not have accurately reflected work-unit exposure over the follow-up period. This analysis only addressed LTSA and did not include SA shorter in length. Also, we did not have sufficient information on employees who changed work-units during follow-up. Should the change in work-unit result in change in LQ level, there is potential for exposure misclassification. However, we speculate any potential misclassification is independent of LTSA during follow-up and that bias would likely attenuate the association toward the null. There is also potential for selection bias in the form of missing data selection bias. However, the results from the sensitivity analysis that includes inverse probability weights suggests that the likelihood of this bias is minimal.

The current study also points towards future research directions that were not within the scope of this study. First, the current study did not examine SA episodes of shorter duration. An earlier longitudinal study of Danish workers reported protective associations between individual-level supervisor support and SA spells of 1–10 days and >10 days for men but not women (37). Whether higher work-unit LQ is more protective against SA spells of shorter length for workers with chronic disorders merits further investigation. Similarly, future research that examines which specific elements of LQ at the work-unit level are of most beneficial influence for workers with chronic disorders may help to inform preventive strategies by occupational healthcare professionals.

### Concluding remarks

In this large sample of Danish public hospital employees, we observed that higher work-unit LQ was associated with lower risk of 1-year LTSA for the large majority of employees including those with chronic somatic disorders. This suggests that LQ is an important work resource for most workers independent of health status. LQ appeared less important for a smaller proportion of employees who suffer from mental disorders, possibly because they already show high rates of LTSA. Future studies should investigate the role of psychosocial resources for increasing labor market participation for workers with and without chronic somatic and mental disorders.

## Supplementary material

Supplementary material
