# Supplementary material for: Chronic disorders, work-unit leadership quality and long-term sickness absence among 33 025 public hospital employees
Source: Scand J Work Environ Health. 2022 Oct 1;48(7):560–8. doi: 10.5271/sjweh.4036 (PMC10539112; doi:10.5271/sjweh.4036)
Supplement: Supplementary material [file SJWEH-48-560-S001.pdf]

Chronic disorders, work-unit leadership quality and long-term sickness absence in 33 025 public hospital employees[1]

by Amar J Mehta, ScD,[2] Jimmi Mathisen, MSc, Tri-Long Nguyen, PhD, Reiner Rugulies, PhD, Naja Hulvej Rod, PhD

1. Supplementary material
2. Correspondence to: Amar Mehta, Department of Public Health, University of Copenhagen, Oster Farimagsgade 5, P.O. Box 2099, 1014 Copenhagen, Denmark. [E-mail: amar.mehta@sund.ku.dk]

Supplementary Figure S1. Study population, exclusion criteria, and follow-up period

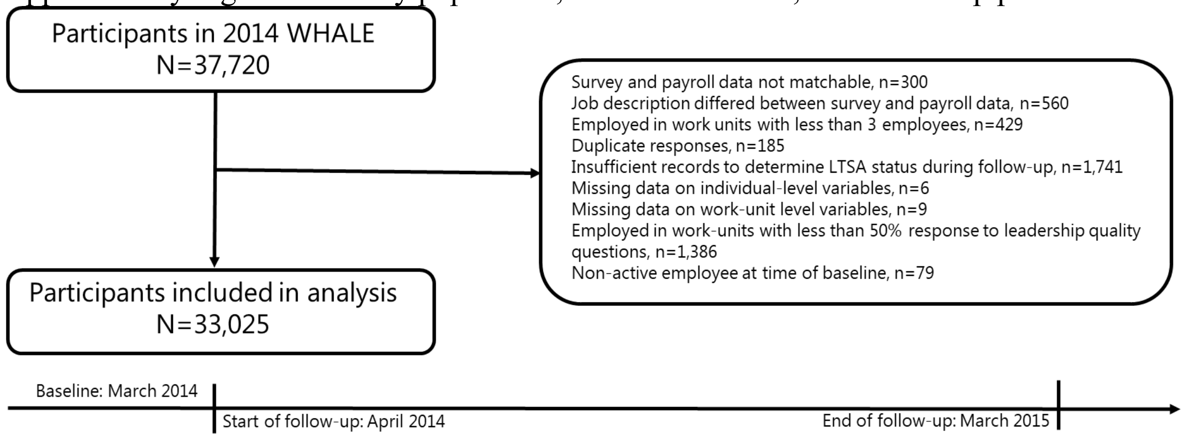

Supplementary Figure S2. Mean leadership quality scores provided by employees in the same work-units

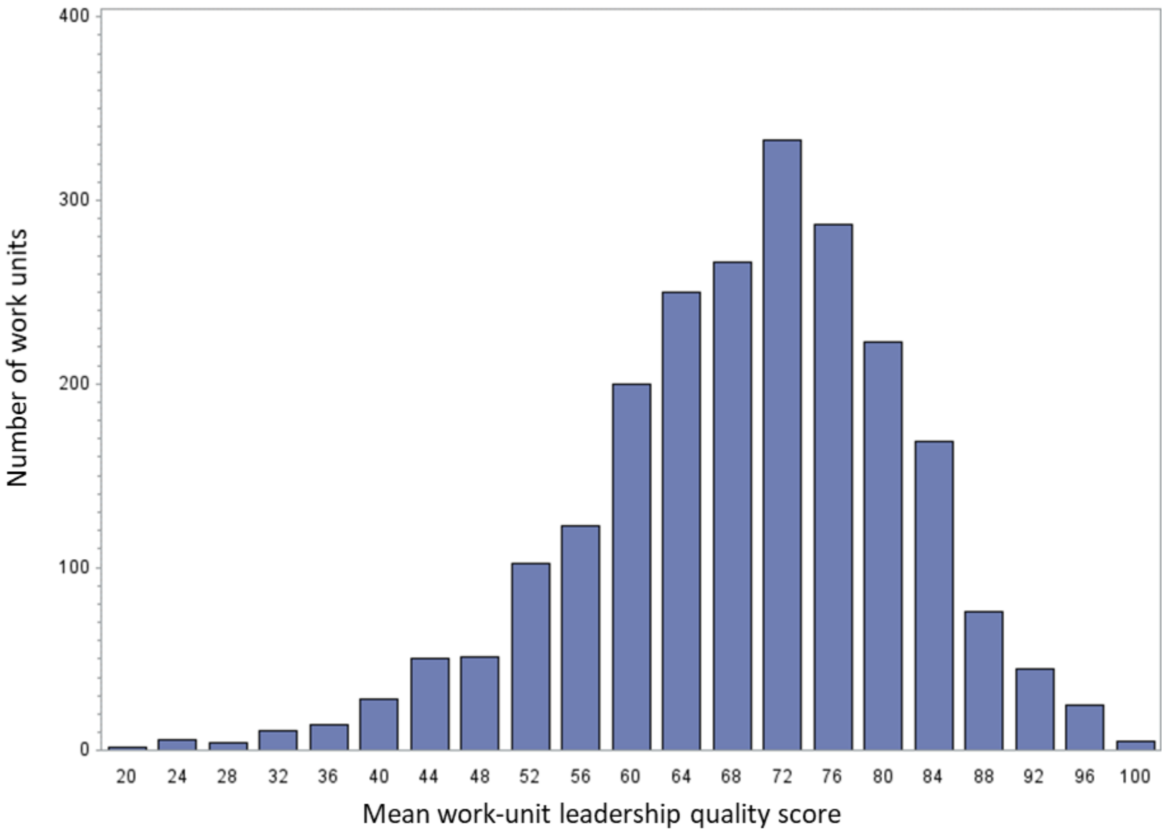

Supplementary Table S1. Overview of included disease groups with associated ICD-10 and ATC codes that were used to define the major chronic disorder subgroupings

[a] International Classification of Diseases-10th edition

[b] Anatomic Therapeutic Chemical Classification System (ATC) codes

DISEASE GROUPS	ICD10[a] DIAGNOSIS CODE	ATC[b] CODE
CHRONIC INFECTION	A52, B18, B20-B24	J05AE-AG;J05AR;J05AB04;J05AX07
CANCER	C00-14;C15-26;C30-34;C43;C45-58;C60-85;C88;C90-97;D45-47	
BLOOD DISEASES	D50-53;D55-D58;D66-68;D69.1;D63.3;D81-84;D89	B03XA
ENDOCRINOLOGICAL DISEASES	E00-03;E05-07;E10-14;E20-27;E31-35;E40-46;E50-E64;E89.0	H03AA01;H03BA02;H03BB01-02;A10A-10B;A10BA02
NEUROLOGICAL DISEASES	G10-14;G20-26;G30-32;G35-37;G40-41;G43-46;G60-G64;G70-73;G80-83;G90-99	N04A-04B;N03AF;N03AX;N02C;N07CA03
THE EYE AND EAR DISEASES	H20-22;H25-28;H30-36;H40;H06.2;H54;H80-91;H93.1;Q16	
CARDIOVASCULAR DISEASES	I05-15;I20-28;I34-50;I60-74;I77;I79	B01AC04;C01AA;C01BC-BD;C01D;C02CA;C07-09
CHRONIC LUNG DISEASES	J41-47;J60-67;J68.4;J70.1;J70.3;J84.1;J84.8-84.9;J96;E84	R03;VO3AN01
DISEASES OF THE DIGESTIVE SYSTEM	K50-52;K57;K71.3-71.9;K72-74;K75.3-75.4;K76;K86;I85;I86.4;I98.2	A07EA;A07EC02-04
SKIN DISEASES	L23-27;L40-41;L43;L45;L93-95;L97	
MUSCULOSKELETAL DISEASES	D86;M02-3;M05-10;M14;M20-25;M30-36;M40-51;M54.3-54.5;M72.6;M75.0;M76;M79.7;M80-85;M84.1;M86.3-86.5;M87-88;M90.0;M95-96;M99	M01AX;M01CB01;M01CB03;L04AA13;A07EC01;M04AA01;M04AB-AC;G03XC01;H05AA03;H05AA02;M05BA
CHRONIC PAIN		M01A;M01AB-AC;M01AE;M01AH;N02AA01-05;N02AA08-10;N02AA51;N02AA55;N02AA58-59;N02AA79;N02AB;N02AB01-03;N02AB52;N02AB72;N02AC;N02AC01-04;N02AX01-03;N02AX05-06;N02AX52;N02B
KIDNEY DISEASES	E85;N03-05;N07-08;N11;N13.0-13.3;N14;N16-19;N25-26;N30.1;N30.4;N31;N32.1-32.2;N36.0;N41.1	
ABDOMINAL DISEASES	N71.1;N73.1;N80	
MENTAL ILLNESSES	F00;G30;G31.1;F01-F04;F06-09;F10.1-10.2;F10.8-10.9;F11.1;F11.5;F11.7-11.9;F12.1-12.2;F12.7-12.9;F13.1-13.2;F13.7-13.9;F14.1-14.2;F14.7-14.9;F15.1-15.2;F15.7-15.9;F16.1-16.2;F16.7-16.9;F18.2;F18.6-18.8;F19.1-19.2;F19.6-19.7;F20-22;F25;F29;F31;F33-34;F40-42;F43.1;F44-45;F50;F60-63;F68-69;K70	N05AN;N06A;N05BB;N05BB;N05BE;N06D;NO7BB01;NO7BB04;NO7BC01-02;NO7BC51

Supplementary Table S2. Distribution of individual and work-unit characteristics between analysis sample and those excluded on basis of missing outcome or exposure

	Excluded sample[a]	Included sample
	**n=2,408**	**n=33,025**
Age, yrs, mean (SD)	42.9 (11.6)	45.6 (11.3)
Female, %	68.9	77.8
Part-time, %	30.0	36.7
Seniority, yrs, mean (SD)	7.6 (8.6)	10.6 (10.0)
Occupational group		
Doctors and dentists	31.4	11.8
Nurses and nursing assistants	29.4	40.9
Other healthcare	10.9	15.5
Education-related staff	2.7	2.4
Service and IT	10.3	11.0
Administrative staff	15.2	18.4
Work-unit size, mean (SD)	24.1 (17.2)	25.6 (19.4)
% Work-unit female, mean (SD)	69.0 (29.0)	77.7 (25.6)
% Work-unit part-time, mean (SD)	29.1 (29.4)	35.8 (28.6)

[a] Excluded sample is subgroup of total excluded sample have missing data on LTSA follow-up status and work-unit leadership quality score

Supplementary Table S3. Distribution of individual and work-unit level characteristics at baseline by chronic disorder subgroup

	**Healthy**	**Only somatic disorder**	**Only mental disorder**	**Both mental and somatic disorder**
N employees	**N=20,081**	**N=10,443**	**N=1,090**	**N=1,411**
Median work-unit leadership quality score (IQR)	68.9 (60.3, 75.7)	69.3 (60.5, 76.2)	68.8 (59.4, 75.2)	68.3 (59.8, 75.6)
Prior long-term sickness (12 months)	2.7	7.6	8.2	12.8
Long-term sickness absence during follow-up (12 months)	4.3	7.6	10.0	14.8
Female, %	76.7	78.7	81.6	83.0
Part-time, %	34.8	37.9	42.8	49.3
Job classification, %				
Doctors and dentists	11.7	12.7	7.3	10.3
Nurses and nursing assistants	41.2	40.0	46.2	38.8
Other healthcare	16.4	14.3	15.6	12.0
Education-related staff	2.4	2.2	2.7	2.5
Service and information technology	10.5	11.8	10.7	11.5
Administrative staff	17.7	19.0	17.6	25.0
Age, years, mean (SD)	43.9 (11.2)	48.7 (11.0)	42.6 (10.3)	48.7 (10.3)
Seniority, years, mean (SD)	9.9 (9.5)	12.3 (10.7)	8.0 (8.1)	10.5 (10.9)
Work-unit size, mean (SD)	25.8 (19.7)	25.0 (18.5)	26.9 (20.0)	26.1 (20.2)
% Work-unit female, mean (SD)	77.4 (25.7)	77.4 (25.9)	80.9 (23.9)	80.7 (24.3)
% Work-unit part-time, mean (SD)	35.5 (28.6)	35.3 (28.6)	40.9 (27.2)	39.5 (27.8)

IQR – interquartile range; SD – standard deviation

Supplementary Table S4. Prevalence of specific chronic disorders in the population

**Chronic disorder type**[a]	**n (%)**
Mental disorders	2,501 (7.6)
Musculoskeletal disorders	4,592 (13.9)
Chronic pain	2,940 (8.9)
Neurological diseases	1,956 (5.9)
Digestive diseases	515 (1.6)
Obstructive lung diseases	1,738 (5.3)
Endocrinological diseases	2,057 (6.2)
Cardiovascular diseases	4,069 (12.3)
Cancer	472 (1.4)
Eye and ear diseases	1,017 (3.1)
Skin diseases	311 (0.9)
Abdominal diseases	138 (0.4)
Kidney diseases	97 (0.3)

[a] Categories for chronic illness type were not mutually exclusive

Supplementary Table S5. Associations[a] between work-unit leadership quality and the risk of individual-level long-term sickness absence during 1-year follow-up after stratification by job classification

**Job classification**	**Doctors and dentists**		**Nurses and nurse assistants**		**Other healthcare and other**		**Education-related staff**		**Service and information technology**		**Administrative staff**	
**Sample size**	**n=3,905**		**n=13,499**		**n=5,130**		**n=782**		**n=3,628**		**n=6,081**	
**Work-unit leadership quality**	**OR**	**95%CI**	**OR**	**95%CI**	**OR**	**95%CI**	**OR**	**95%CI**	**OR**	**95%CI**	**OR**	**95%CI**
Tertile 1 (Low)	1.00	---	1.00	---	1.00	---	1.00	---	1.00	---	1.00	---
Tertile 2 (Medium)	0.84	0.53-1.32	0.85	0.72-0.99	0.86	0.64-1.17	0.38	0.19-0.75	0.92	0.68-1.25	0.94	0.72-1.23
Tertile 3 (High)	0.66	0.41-1.07	0.74	0.63-0.88	0.78	0.56-1.08	0.29[b]	0.14-0.61	0.74	0.54-1.00	0.82	0.62-1.08

[a] As estimated in multilevel logistic regression with random intercept for work-unit and includes adjustment for individual-level age, sex, part-time status, job seniority, and job classification, and work-unit level unit size, proportion of female employees in work-unit, and proportion of part-time employees in work-unit

[b] Pair-wise multiplicative interaction between high tertile work-unit leadership quality and education-related staff was statistically significant ($p<0.05$)

Supplementary Table S6. Associations between work-unit leadership quality and individual-level long-term sickness absence during 1-year follow-up with and without adjustment for household income and marital status

	**Multiple adjusted[a]**		**Multiple adjusted + household income and marital status[b]**	
	**OR**	**95%CI**	**OR**	**95%CI**
**Work-unit leadership quality**				
Tertile 1 (Low)	1.00	---	1.00	---
Tertile 2 (Medium)	0.84	0.76-0.94	0.85	0.76-0.95
Tertile 3 (High)	0.73	0.65-0.82	0.74	0.69-0.83

[a] As estimated in multilevel logistic regression with random intercept for work-unit and adjusted for age, sex, and individual-level part-time status, job seniority, and job classification, and work-unit level unit size, proportion of female employees in work-unit, and proportion of part-time employees in work-unit
[b] Multiple adjusted model includes includes additional adjustment for household income (quartiles) and marital status (binary, yes/no)

Supplementary Table S7. Associations between work-unit leadership quality and risk of individual-level long-term sickness absence during 1-year follow-up with and without inverse probability weighting for exclusion on basis of missing exposure or outcome

	**Multiple adjusted**[a]		**Multiple adjusted + IPW**[b]	
	**OR**	**95%CI**	**OR**	**95%CI**
**Work-unit leadership quality**				
Tertile 1 (Low)	*Ref*	---	*Ref*	---
Tertile 2 (Medium)	0.84	0.76-0.94	0.82	0.74-0.92
Tertile 3 (High)	0.73	0.65-0.82	0.72	0.64-0.80

[a] As estimated in multilevel logistic regression with random intercept for work-unit and adjusted for age, sex, and individual-level part-time status, job seniority, and job classification, and work-unit level unit size, proportion of female employees, and proportion of part-time employees

[b] Multiple adjusted model includes inverse probability weighting for exclusion from the study sample due to missing LTSA during follow-up or work-unit leadership quality
